# Does the awareness of having a lumbar spondylolisthesis influence self-efficacy and kinesiophobia? A retrospective analysis

**DOI:** 10.1186/s40945-019-0070-7

**Published:** 2019-12-16

**Authors:** Silvano Ferrari, Rosa Striano, Eric Lucking, Paolo Pillastrini, Marco Monticone, Carla Vanti

**Affiliations:** 1 Department of Biomedical Sciences, University of Padova, Padova, Italy; 2 Progetto Persona Onlus, Korian Group, Milan, Italy; 3 CRU International, London, UK; 4 Department of Biomedical and Neuromotor Sciences (DIBINEM), Alma Mater Studiorum, University of Bologna, Bologna, Italy; 5 Department of Medical Sciences and Public Health, University of Cagliari, Cagliari, Italy

**Keywords:** Spondylolisthesis, Pain self-efficacy, Fear of movement, Spinal pain, Outcome measures

## Abstract

**Background:**

High pain self-efficacy and low kinesiophobia seem related to a better prognosis in patients complaining of low back pain (LBP). The literature stresses the potential negative effects of anatomical defect diagnosis (e.g. lumbar spondylolisthesis) on the psychological profile. The aim of this study is to investigate the relationships between awareness of having a spondylolisthesis, pain self-efficacy and kinesiophobia.

**Methods:**

A secondary retrospective analysis was done. Ninety-eight subjects with subacute and chronic LBP were included: 49 subjects with diagnosed symptomatic lumbar spondylolisthesis and 49 subjects with diagnosed non-specific LBP. The pain self-efficacy measured with the Pain Self-Efficacy Questionnaire and the fear of movement measured with the Tampa Scale of Kinesiophobia were considered variables to investigate, whereas diagnosis and demographic/clinical variables were considered predictors or potential confounders.

**Results:**

By comparing the two groups, the awareness of having a spondylolisthesis did not significantly influence neither pain self-efficacy (*p* = 0.82), nor kinesiophobia (*p* = 0.75). Higher perceived pain reduces pain self-efficacy and increases kinesiophobia in both groups (*p* = 0.002 and p = 0,031 respectively).

**Conclusions:**

It seems that the awareness of an anatomical defect as spondylolisthesis does not significantly affect the beliefs of carry out activities and movements despite the pain. Other studies with wider samples are required, to confirm these preliminary results.

## Introduction

Low back pain (LBP) is one of the most important causes of disability worldwide and is associated with enormous social and economic costs [1]. LBP is usually defined as “non-specific” when no anatomical or pathological cause can be recognized to explain pain, whereas it is considered “specific” in case of well-identified reasons for pain, e.g. osteoporosis with fracture, or pathological conditions as inflammation, infection, and so on [1]. Specific causes account for less than 20% of the cases of LBP [2].

Spondylolisthesis is defined as the slipping of a vertebral body in relation to the one below and is classified in isthmic and degenerative [3]. The gold standard for diagnosis is the imaging (Dynamic X-rays, Computed Tomography, Magnetic Resonance), since history and clinical exams do not show an acceptable diagnostic performance [4].

In fact, most signs in symptomatic spondylolisthesis are comparable with those of non-specific LBP, not allowing the clinician to make differential diagnosis by the clinical examination itself [5]. The only clinical signs typical of spondylolisthesis seem to be “Low Midline Sill Sign Test” for lumbar spondylolisthesis and the “Interspinous Gap Change Test” for Lumbar Instability [6]. Moreover, a strict association between spondylolisthesis and LBP was not been confirmed in epidemiological studies on the general adult population [7].

It is known that pain is not only a direct consequence of pathology or anatomic lesion but is influenced by some psychological factors. Pain seems to be the result of an individual past experiences of pain and healthcare, as well as cultural and educational characteristics. High pain self-efficacy and low fear of movement are considered relevant in reducing the consequences of LBP in terms of catastrophizing and depression [8]. Attitudes, beliefs and behaviours may also play a key role in recovery from chronic symptoms [9]. Patients with chronic LBP more strongly endorse “anatomic” pain beliefs compared to pain-free individuals and commonly transpose same beliefs of their healthcare professionals and select treatments that are consistent with their own beliefs [10].

Beliefs of pain due to anatomical lesion may focus the subject on negative concepts of damage, illness and harm may be associated to greater fear-avoidance beliefs and correspondingly higher disability [11–13]. Fear-avoidance beliefs and kinesiophobia concern the avoidance of activities due to the beliefs that they will cause pain or damage [14, 15]. Other beliefs include those of self-efficacy (the functioning despite pain) [16], and beliefs about control, for example that pain can be controlled by oneself (internal) or by other conditions (external).

Pain self-efficacy may depend on three factors: experience, observation of others with similar problems, and education [17]. Poor self-efficacy is associated with pain intensity and disability [18], interferes with the recovery, and predicts long-term disability [19]. Therefore, increased pain catastrophizing, fear of movement, and passive coping due to an excessive patient attention to some anatomical bases for his/her LBP could negatively influence the possibility of recovery [20].

Several authors suggest avoiding communication with patients by “anatomical diagnosis”, posing instead higher stress on the favorable prognosis of LBP, the importance of having active coping strategies, the explanation of pain mechanisms, etc. [21]. In the absence of radicular pain, spondylolisthesis is usually considered a sort of non-specific LBP; however, from the patient perspective the awareness of having a vertebral slipping may potentially influence both pain self-efficacy and kinesiophobia [22]. Nevertheless, no previous studies confirmed the hypothesis that individual self-efficacy and/or kinesiophobia can change in subjects who have received an explicit diagnosis of symptomatic lumbar spondylolisthesis.

The main aim of this study is to investigate the relationships between awareness of having a spondylolisthesis, pain self-efficacy and kinesiophobia, by comparing two different samples of LBP patients: patients diagnosed with spondylolisthesis, and patients diagnosed with non-specific LBP. Our assumption was that the awareness of having an “anatomical failure”, obtained after seeing x-ray imaging and/or after receiving medical diagnosis of spondylolisthesis, could lead to higher kinesiophobia and less self-efficacy, together with higher disability levels. Our hypothesis was that patients with spondylolisthesis, who know they have a slipped vertebra that it could not come back, may have less self-efficacy and more fear-avoidance behaviors, compared to patients with non-specific LBP.

Moreover, we aimed to investigate the relationships between main socio-demographical and clinical characteristics, and psychological patient profile.

## Methods

The STROBE recommendations for observational studies were followed [23].

### Study design

Monocentric retrospective analysis.

### Setting

The medical records of 100 consecutive outpatients with subacute or chronic LBP submitted to a conservative treatment in a physiotherapy clinic located in northern Italy were retrospectively reviewed. More specifically, these records were already used for two previous studies [24, 25].

#### Population

The eligible patients for this secondary analysis were 223 subjects (120 with spondylolisthesis diagnosis from the study by Vanti et al., 2017, and 103 with non-specific LBP diagnosis from the study by Ferrari et al., 2016). First, we selected the records coming from only one physical therapy clinic and collected by the same physical therapist (to avoid bias related to different way of communication with patients), leaving a sample of 53 outpatients with spondylolisthesis and 49 with non-specific LBP. To compare two groups with equal size, we deleted the last three records from the Vanti et al. spreadsheet. Therefore, this secondary analysis included 98 outpatients with subacute or chronic LBP (49 with diagnosed symptomatic lumbar spondylolisthesis, and 49 with diagnosed non-specific LBP), who were retrospectively studied (Ferrari et al., 2016; Vanti et al., 2017). Inclusion criteria concerned outpatients older than 18 years, complaining of LBP lasting for at least 1 month, with or without referred pain. Exclusion criteria were previous lumbar surgery, systemic diseases (inflammation, infection, cancer, etc.), neuromuscular disorders or cognitive deficits.

This sample was composed of women (59%) and men (41%), with a mean age of 49 years and a LBP mean duration of 14 months. Socio-demographical and clinical characteristics and scales scores (PSEQ-I, TSK-I, and NRS) were collected in a MS Excel spreadsheet.

#### Measures

All participants filled in the PSEQ-I, the TSK-I, and the NRS at the starting of their treatment period and after having received their clinical diagnosis, that was symptomatic lumbar spondylolisthesis, or non-specific LBP.

The PSEQ-I was used to measure the degree of pain self-efficacy [26]. Each patient was asked to rate how confidently he/she can perform some activities despite his/her pain. Each item is scored on a 7-point Likert scale, where 0 = not at all confident, and 6 = completely confident. Total scores are calculated by adding the score of each item, ranging from 0 to 60. Higher scores reflect stronger self-efficacy beliefs, whereas low scores indicate a subject more focused on his/her pain. PSEQ-I scores higher than 40 indicate a subject who is well responding to an exercise program [27] or sustaining and building on one‘s own functional gains [16]. The Italian version of the PSEQ (PSEQ-I) showed to be unidimensional, to display good internal consistency, reliability and construct validity, to have no floor/ceiling effects [26], and good responsiveness [28].

The TSK-I aimed to measure the beliefs in relation to pain and physical activities. It is composed of 13 items ranging from 1 = completely disagree, to 4 = completely agree, with total score from 13 to 52 [29]. It is divided in two subscales: TSK 1 – Activity Avoidance, and TSK 2 – Harm. The kinesiophobia is considered relevant if the TSK total score is ≥37 [30]. The Tampa Scale of Kinesiophobia (TSK) showed good reliability and validity, also in its Italian Version (TSK-I) [30].

Pain intensity (subjective perception of pain) is usually measured with a Numerical Rating Scale (NRS), which demonstrated good sensitivity and responsiveness [31].

It is composed of 11 values ranging from 0 (no pain) to 10 (unbearable pain), indicating the main intensity of pain experienced by a patient in the last week. A significant relationship between changes on the Pain Intensity and the Patient Global Impression of Change was demonstrated [32].

Pain self-efficacy measured with the PSEQ-I and fear of movement measured with the TSK-I were considered the variables to investigate, and assume the role of response variables, whereas the other variables (clinical diagnosis, pain characteristics, psychosocial variables, etc.) were considered predictors or potential confounders. One research assistant provided all the participants with written information concerning the questionnaires and procedures.

#### Ethical considerations

Based on the study design, Institutional Review Board approvals were previously obtained, consent forms had already been signed, and further forms were not required. The privacy rights of participants were observed, and the procedures followed were in accordance with Italian ethical standards and with the Helsinki Declaration of 1975, as revised in 2000.

#### Statistical analysis

Given the retrospective nature of the study, a post-hoc power analysis was done to avoid underpowered results at the time of the final statistical analysis.

All continuous variables were summarized using descriptive statistics and in particular were reported as mean ± standard deviation (SD). Categorical or dichotomous variables were reported with their absolute and relative frequencies. Linear regression analysis was adopted to explore the correlation between the PSEQ-I, TSK-I, and socio-demographic and clinical characteristics.

To analyze in a deeper way the results in relation to the level of self-efficacy, we stratified our sample in two different subgroups: high self-efficacy (PSEQ-I score > 40) and low self-efficacy (PSEQ-I score ≤ 40). Similarly, for kinesiophobia we stratified our sample in two subgroups: high kinesiophobia (TSK ≥ 37) and low kinesiophobia (TSK < 37).

All tests were considered significant with *p* values less than 0.05 and 95% confidence intervals were provided for variables subjected to statistical inference. All statistical analyses were performed using STATA, version 15.1.

## Results

### Participants

The medical records of 98 consecutive outpatients with subacute or chronic LBP (49 diagnosed by having lumbar symptomatic spondylolisthesis, 49 diagnosed by having non-specific LBP) afferent to one physical therapy clinic were retrospectively screened. Student’s t-test did not show any relevant differences in baseline characteristics between groups.

### Descriptive data

Table 1 shows the socio-demographic and clinical characteristics of the sample, whereas Table 2 illustrates mean and SD of pain self-efficacy, amount of pain and kinesiophobia. Sixty-four subjects (74.5% of the sample) did not reach 40 points PSEQ-I score, showing poor self-efficacy, and 68 subjects (69.4% of the sample) reached 37 TSK-I point score, showing high fear of movement.

**Table 1:** Socio-demographic characteristics of the study population (*n* = 98)

Variable		%
Gender		
Female		59
Male		41
Married		
Yes		64,3
No		35,7
Education		
Elementary school		0
Middle school		5,1
High school		45,9
University		49
Smoking		
Yes		34,7
No		65,3
Pain duration (mean – sd)	14 (months)	12,37
Drugs		
No		53
Yes (NSAIDs, pain killers, etc)		47

**Table 2:** Rating scales of the study population (*n* = 98)

Variable	No.	%
Pain self-efficacy		
PSEQ-I (mean – sd)	30.44 ± 14.11	
PSEQ-I > 40	23	23.47%
PSEQ-I ≤ 40	75	76.53%
Pain		
NRS (mean - sd)	48.56 ± 17.57	
NRS 0 = no Pain	0	0
NRS 1–3 = Mild Pain	26	26.53%
NRS 4–6 = Moderate Pain	52	53.06%
NRS 7–10 = Severe Pain	20	20.41%
Kinesiophobia		
TSK-I (mean - sd))	32.9 ± 7.53	
TSK-I-1 Activity Avoidance (mean – sd)	41.38 ± 3.44	
TSK-I-2 Harm (mean – sd)	28.36 ± 4.66	
TSK-I > 37	30	30.61%
TSK-I ≤ 37	68	69.39%

*NRS* Numerical Rating Scale

*PSEQ-I* Pain Self-Efficacy Questionnaire – Italian version

*TSK-I* Tampa Scale ok Kinesiophobia – Italian version

### Outcome data and main results

Post-hoc power analysis based on effect size = 0.61, α = 0.05, and sample size = 98 demonstrated high power of the present study (1-β error probability = 1.000). The relationships between PSEQ-I, TSK-I and the following demographic and clinical characteristics were investigated: diagnosis, age, sex, marital status, educational level, smoking, amount of pain, duration of pain, and use of drugs. Concerning the influence of these characteristics on psychological patient profile, linear regression showed that having received a diagnosis of symptomatic lumbar spondylolisthesis does not influence neither pain self-efficacy (*p* = 0.82), nor kinesiophobia (*p* = 0.75), compared with patients having received a diagnosis of non-specific LBP. Moreover, low PSEQ-I and high TSK-I rates appeared significantly related to higher pain intensity (*p* = 0.002, and *p* = 0,031 respectively).

High educational level (university degree) was borderline related (*p* = 0.06) with higher self-efficacy, but not significantly related with lower kinesiophobia (*p* = 0.12). No other demographic or clinical characteristics significantly modified self-efficacy or kinesiophobia in this sample. Moreover, higher self-efficacy levels were significantly associated to lower kinesiophobia (R = -0,53). After having stratified the subjects for high versus low levels of self-efficacy and kinesiophobia, these results did not significantly change in both groups.

## Discussion

This study aimed to investigate whether to be aware of having an “anatomical failure” (symptomatic lumbar spondylolisthesis) may modify pain self-efficacy and kinesiophobia, in subjects complained of subacute or chronic LBP. Our assumption was that the awareness of permanent damage and potentially chronic pain could lead to modification of beliefs with passivity, inactivity, pain-avoidance, all of which may provoke kinesiophobia and less self-efficacy, with higher disability in the future. This consideration of damage about your own spine could also be enhanced by practitioner or spine surgeon explanations about the diagnosis and related complications. Some studies confirmed that not verbal communication, the words used for the diagnosis and the received prescriptions could influence the patient’s thoughts and believes after a physician visit [33–35]. Despite guideline recommendations on this topic [36, 37], the attitude and behaviour of health care practitioners are not always coherent, consequently, patients receive different inputs and develop different believes, thoughts and behaviours [38].

The second aim of this study was to investigate the relationship between some demographic and clinical characteristics, and pain self-efficacy and kinesiophobia.

The results of the present study did not confirm our expectations, which were based on the current literature on catastrophizing and fear of movement. In fact, we cannot confirm any significant correlation between the awareness of having a spondylolisthesis, self-efficacy and kinesiophobia. Having received an “anatomical defect diagnosis”, like spondylolisthesis, does not correspond to an excessive patient “attention”, which can affect the way in which a subject handles their thoughts, pain, and daily living activities.

Current literature considers diagnostic labelling referred to anatomical basis for LBP, frequently endorsed by healthcare professionals, as significantly influencing patients’ beliefs [39], especially for some diagnoses. For example, patients with fibromyalgia, a not-inflammatory musculoskeletal pain syndrome, more strongly endorsed catastrophizing beliefs than patients with rheumatoid arthritis [40], and Individuals with osteoporosis have higher levels of kinesiophobia compared to healthy subjects of the same sex and age [41].

Moreover, many studies showed that disc surgery patients are at a higher risk of suffering from depression and anxiety than the general population. These studies emphasized the relevance for clinicians to consider psychological concerns in patients undergoing disc surgery [42, 43].

At the best of the authors’ knowledge, no previous similar study was performed on beliefs and behaviours in lumbar spondylolisthesis. However, some studies showed that pain self-efficacy and lumbar function may improve in symptomatic lumbar spondylolisthesis through a rehabilitation program centred on cognitive and behavioural principles, both in conservative setting [18], and after surgical fusion [44, 45].

A possible explanation for the results of the present study comes from a qualitative study conducted on chronic pain patients with high kinesiophobia [46]. One of the most relevant themes emerged from patients in that research was the seeking of a diagnostic certainty from health practitioners, to make sense of their pain. Patients receiving diagnostic uncertainty, or diagnosis of an underlying pathology that could not be confirmed, are more confused and fearful [46]. Therefore, the awareness of having received a clear diagnosis may have counterbalanced the negative influence of that diagnosis on pain self-efficacy and kinesiophobia.

For patients with chronic pain, more than the diagnosis “per se*”*, the fully understanding of the diagnostic jargon and the implications of diagnostic label could be relevant. Among patients with fibromyalgia, who do not make sense to their symptoms will more likely catastrophize [47]. Moreover, not only individual diagnosis, but also response patterns of pain, psychological processing, and information processing are involved in exacerbation and maintenance of chronic pain [48].

The second result of the present study concerns the relationship between higher pain, reduced pain self-efficacy and increased kinesiophobia in both groups. This finding is coherent to other studies on chronic LBP, showing that pain severity is related to high Fear Avoidance Beliefs Questionnaires scores [49, 50].

Concerning self-efficacy, there is evidence that high level of self-efficacy correlates to lower intensity of pain and disability, in patients with chronic pain [51–53]. On the contrary, poor self-efficacy and fear of movement are related to an increased level of disability [54] and low physical activity [55–57]. The authors of these studies think that these factors may be related to pain.

Concerning demographic characteristics, we did not find any relevant correlations between psychological profile, age and gender, according to the results of Rahman and colleagues [58] on chronic musculoskeletal pain patients. Moreover, in the present study, pain self-efficacy and kinesiophobia were not related to the older age, according to Wettstein and colleagues [59]. In agreement with a previous study [58], we did not find any significant correlation between educational level, pain self-efficacy and kinesiophobia. Finally, we did not find any significant relationship with smoking, despite it appears associated to higher pain intensity and sedentary lifestyle [60].

Our study is the first one investigating the relationship between pain self-efficacy, kinesiophobia and clinical diagnosis of spondylolisthesis. The relevance of our study is the investigation of main characteristics potentially influencing the psychological patient profile in lumbar spondylolisthesis.

The results of the present study did not confirm any significant correlation between the awareness of having a spondylolisthesis, and self-efficacy and kinesiophobia. No significant correlation has also been found between the awareness of having a spondylolisthesis and the patient’s sociodemographic characteristics. Only higher pain levels were significantly related to a worse psychological condition.

The main limitation of this study is the generalization of the results, due to the characteristics of our sample, composed of subjects with a medium-high level of instruction, residents in northern Italy. Moreover, the dimension of the sample may have influenced the results. Further studies on higher samples are suggested on the relationship between medical diagnosis, self-efficacy and fear of movement, both in spondylolisthesis and in other clinical conditions.

Another limitation of our study concerns the lack of data about the time elapsed between the diagnoses of spondylolisthesis received by a physician or surgeon and the moment in which patients filled the questionnaires. Consequently, we could not investigate the influence of the time on the beliefs about the presence of spondylolisthesis.

## Conclusions

This study showed that in Italian population complained of subacute and chronic LBP, the awareness of an anatomical defect as spondylolisthesis does not significantly affect the beliefs in carrying out activities and movements despite the pain. Conversely, the amount of pain seems significantly influence the psychological patient profile.

From a clinical point of view, the knowledge of main characteristics potentially influencing the patient perspective on his/her condition may be useful to better manage the clinician communication and patient education.

## Data Availability

The datasets used and/or analysed during the current study are available from the corresponding author on reasonable request.

## References

[1] Hartvigsen Jan, Hancock Mark J, Kongsted Alice, Louw Quinette, Ferreira Manuela L, Genevay Stéphane, Hoy Damian, Karppinen Jaro, Pransky Glenn, Sieper Joachim, Smeets Rob J, Underwood Martin, Buchbinder Rachelle, Hartvigsen Jan, Cherkin Dan, Foster Nadine E, Maher Chris G, Underwood Martin, van Tulder Maurits, Anema Johannes R, Chou Roger, Cohen Stephen P, Menezes Costa Lucíola, Croft Peter, Ferreira Manuela, Ferreira Paulo H, Fritz Julie M, Genevay Stéphane, Gross Douglas P, Hancock Mark J, Hoy Damian, Karppinen Jaro, Koes Bart W, Kongsted Alice, Louw Quinette, Öberg Birgitta, Peul Wilco C, Pransky Glenn, Schoene Mark, Sieper Joachim, Smeets Rob J, Turner Judith A, Woolf Anthony (2018). What low back pain is and why we need to pay attention. The Lancet.

[2] Ehrich GE (2003). Low back pain. Bull World Health Organ.

[3] Koreckij TD, Fischgrund JS (2015). Degenerative spondylolisthesis. J Spinal Disord Tech.

[4] Jarvik JG, Deyo RA (2002). Diagnostic evaluation of low back pain with emphasis on imaging. Ann Intern Med.

[5] Petersen T, Laslett M, Juhl C (2017). Clinical classification in low back pain: best-evidence diagnostic rules based on systematic reviews. BMC Musculoskelet Disord.

[6] Ahn, Jhun (2015). New physical examination tests for lumbar spondylolisthesis and disability: low midline sign and interspinous gap change during lumbar flexion-extension motion. BMC Musculoskelet Disord.

[7] Andrade NS, Ashton CM, Wray NP, Brown C, Bartanusz V (2015). Systematic review of observational studies reveals no association between low back pain and lumbar spondylolysis with or without isthmic spondylolisthesis. Eur Spine J.

[8] Cheng ST, Leung CMC, Chan KL, Chen PP, Chow YF, Chung JWY (2018). The relationship of self-efficacy to catastrophizing and depressive symptoms in community-dwelling older adults with chronic pain: a moderated mediation model. PLoS One.

[9] Hasenbring MI, Verbunt JA (2010). Fear-avoidance and endurance-related responses to pain: new models of behaviour and their consequences for clinical practice. Clin J Pain.

[10] Werner EL, Ihlebaek C, Skouen JS, Laerum E (2005). Beliefs about low back pain in the Norwegian general population are they related to pain experiences and health professionals?. Spine.

[11] Walsh DA, Radcliffe JC (2002). Pain beliefs and perceived physical disability of patients with chronic low back pain. Pain.

[12] Roelofs J, Goubert L, Peters ML, Vlaeyen JW, Crombez G (2004). The Tampa scale for kinesiophobia: further examination of psychometric properties in patients with chronic low back pain and fibromyalgia. Eur J Pain.

[13] Sloan TJ, Gupta R, Zhang W, Walsh DA (2008). Beliefs about the causes and consequences of pain in patients with chronic inflammatory or noninflammatory low Back pain and in pain-free individuals. Spine.

[14] Waddell G, Newton M, Henderson I, Somerville D, Main CJ (1993). A fear-avoidance beliefs questionnaire (FABQ) and the role of fear-avoidance beliefs in chronic low back pain disability. Pain.

[15] Vlaeyen JW, Kole-Snijders AM, Boeren RG, van Eek H (1995). Fear of movement/(re) injury in chronic low back pain and its relation to behavioural performance. Pain.

[16] Nicholas MK (2007). The pain self-efficacy questionnaire: taking pain into account. Eur J Pain.

[17] Tonkin L (2008). The pain self-effcacy questionnaire. Aust J Physiother.

[18] Ferrari S, Vanti C, Costa F, Fornari M (2016). Can physical therapy centred on cognitive and behavioural principles improve pain self-efficacy in symptomatic lumbar isthmic spondylolisthesis? A case series. J Bodyw Mov Ther.

[19] Bandura A (1977). Self-efficacy: toward a unifying theory of behavioral change. Psychol Rev.

[20] van Ravesteijn H, van Dijk I, Darmon D, van de Laar F, Lucassen P, Hartman TO (2012). The reassuring value of diagnostic tests: a systematic review. Patient Educ Couns.

[21] van den Bosch MA, Hollingworth W, Kinmonth AL, Dixon AK (2004). Evidence against the use of lumbar spine radiography for low back pain. Clin Radiol.

[22] Ziadni MS, Sturgeon JA, Darnall BD (2018). The relationship between negative metacognitive thoughts, pain catastrophizing and adjustment to chronic pain. Eur J Pain.

[23] von Elm E, Altman DG, Egger M, Pocock SJ, Gøtzsche PC, Vandenbroucke JP (2008). The strengthening the reporting of observational studies in epidemiology (STROBE) statement: guidelines for reporting observational studies. J Clin Epidemiol.

[24] Ferrari S, Chiarotto A, Pellizzer M, Vanti C, Monticone M (2016). Pain self-efficacy and fear of movement are similarly associated with pain intensity and disability in Italian patients with chronic low back pain. Pain Pract.

[25] Vanti Carla, Ferrari Silvano, Berjano Pedro, Villafañe Jorge Hugo, Monticone Marco (2017). Responsiveness of the bridge maneuvers in subjects with symptomatic lumbar spondylolisthesis: A prospective cohort study. Physiotherapy Research International.

[26] Chiarotto A, Vanti C, Ostelo RW, Ferrari S, Tedesco G, Rocca B (2015). The pain self-efficacy questionnaire: cross-cultural adaptation into Italian and assessment of its measurement properties. Pain Pract.

[27] Frost H, Klaber Moffett JA, Moser JS, Fairbank JC (1995). Randomized controlled trial for evaluation of fitness program for patients with chronic low back pain. BMJ.

[28] Chiarotto A, Vanti C, Cedraschi C, Ferrari S, de Lima E Sà Resende F, Ostelo RW, Pillastrini P (2016). Responsiveness and minimal important change of the pain self-efficacy questionnaire and short forms in patients with chronic low back pain. J Pain.

[29] Kori SH, Miller RP, Todd DD (1990). Kinesiophobia: a new view of chronic pain behavior. Pain Management.

[30] Monticone M, Giorgi I, Baiardi P, Barbieri M, Rocca B, Bonezzi C (2010). Development of the Italian version of the Tampa scale of Kinesiophobia (TSK-I): cross-cultural adaptation, factor analysis, reliability and validity. Spine.

[31] Hjermstad MJ, Fayers PM, Haugen DF, Caraceni A, Hanks GW, Loge JH (2011). Studies comparing numerical rating scales, verbal rating scales, and visual analogue scales for assessment of pain intensity in adults: a systematic literature review. J Pain Symptom Manage.

[32] Farrar JT, Young JP, La Moreaux L, Werth JL, Poole RM (2001). Clinical importance of changes in chronic pain intensity measured on an 11-point numerical pain rating scale. Pain.

[33] Houben RM, Ostelo RW, Vlaeyen JW, Wolters PM, Peters M, Stomp-van den Berg SG (2005). Health care providers’ orientations towards common low back pain predict perceived harmfulness of physical activities and recommendations regarding return to normal activity. Eur J Pain.

[34] Ha JF, Longnecker N (2010). Doctor-patient communication: a review. Ochsner J.

[35] Kennedy BM, Rehman M, Johnson WD, Magee MB, Leonard R, Katzmarzyk PT (2017). Healthcare providers versus patients’ understanding of health beliefs and values. Patient Exp J.

[36] Bishop A, Foster NE, Thomas E, Hay EM (2008). How does the self-reported clinical management of patients with low back pain relate to the attitudes and beliefs of health care practitioners? A survey of UK general practitioners and physiotherapists. Pain..

[37] Pincus T, Greenwood L, McHarg E (2011). Advising people with back pain to take time off work: a survey examining the role of private musculoskeletal practitioners in the UK. Pain..

[38] Darlow B, Fullen BM, Dean S, Hurley DA, Baxter GD, Dowell A (2012). The association between health care professional attitudes and beliefs and the attitudes and beliefs, clinical management, and outcomes of patients with low back pain: a systematic review. Eur J Pain.

[39] Martindale J, Smith J, Sutton CJ, Grennan D, Goodacre L, Goodacre JA (2006). Disease and psychological status in ankylosing spondylitis. Rheumatology.

[40] Hassett AL, Cone JD, Patella SJ, Sigal LH (2000). The role of catastrophizing in the pain and depression of women with fibromyalgia syndrome. Arthritis Rheum.

[41] Gunendi Z, Eker D, Tecer D, Karaoglan B, Ozyemisci-taskiran O (2018). Is the word “osteoporosis” a reason for kinesiophobia?. Eur J Phys Rehabil Med.

[42] Zieger M, Schwarz R, König HH, Härter M, Riedel-Heller SG (2010). Depression and anxiety in patients undergoing herniated disc surgery: relevant but under researched - a systematic review. Cen Eur Neurosurg.

[43] Dorow M, Löbner M, Stein J, Konnopka A, Meisel HJ, Günther L (2017). Risk factors for postoperative pain intensity in patients undergoing lumbar disc surgery: a systematic review. PLoS One.

[44] Abbott AD, Tyni-Lenné R, Hedlund R (2010). Early rehabilitation targeting cognition, behavior, and motor function after lumbar fusion: a randomized controlled trial. Spine (Phila Pa 1976).

[45] Monticone M, Ferrante S, Teli M, Rocca B, Foti C, Lovi A (2014). Management of catastrophising and kinesiophobia improves rehabilitation after fusion for lumbar spondylolisthesis and stenosis. A randomized controlled trial. Eur Spine J.

[46] Bunzli S, Smith A, Schütze R, O’Sullivan P (2015). Beliefs underlying pain-related fear and how they evolve: a qualitative investigation in people with chronic back pain and high pain-related fear. BMJ Open.

[47] van Wilgen CP, van Ittersum MW, Kaptein AA, van Wijhe M (2008). Illness perceptions in patients with fibromyalgia and their relationship to quality of life and catastrophizing. Arthritis Rheum.

[48] Rusu AC, Gajsar H, Schlüter MC, Bremer YI (2019). Cognitive biases toward pain: implications for a neurocognitive processing perspective in chronic pain and its interaction with depression. Clin J Pain.

[49] Nava-Bringas TI, Macias-Hernandez IM, Vásquez-Ríos JR, Coronado-Zarco R, Miranda-Duarte R, Cruz-Medina E (2017). Fear-avoidance beliefs increase perception of pain and disability in Mexicans with chronic low back pain. Revista Brasileira de Reumatologia (English Edition).

[50] Wertli MM, Rasmussen-Barr E, Held U, Weiser S, Bachmann LM, Brunner F (2014). Fear-avoidance beliefs—a moderator of treatment efficacy in patients with low back pain: a systematic review. Spine J.

[51] Denison E, Asenlof P, Lindberg P (2004). Self-efficacy, fear avoidance, and pain intensity as predictors of disability in subacute and chronic musculoskeletal pain patients in primary health care. Pain.

[52] Dohnke B, Knauper B, Muller-Fahrnow W (2005). Perceived self-efficacy gained from, and health effects of a rehabilitation program after hip. Arthritis Rheum.

[53] La Touche R, Grande-Alonso M, Arnés-Prieto P, Paris-Alemany A (2019). How does self efficacy influence pain perception, postural stability and range of motion in individuals with chronic low back pain?. Pain Physician.

[54] Brandao de Moraes Vieira E, Goes Salvetti M, Petri Damiani L, Andruciolide Mattos Pimenta C (2014). Self-efficacy and fear avoidance beliefs in chronic low back pain patients: coexistence and associated factors. Pain Manag Nurs.

[55] Bauman AE, Reis RS, Sallis JF, Wells JC, Loos RJ, Martin BW (2012). Correlates of physical activity: why are some people physically active and others not?. Lancet..

[56] Woby SR, Roach NK, Urmston M, Watson PJ (2007). The relation between cognitive factors and levels of pain and disability in chronic low back pain patients presenting for physiotherapy. Eur J Pain.

[57] Lundberg M, Frennered K, Hagg O, Styf J (2011). The impact of fear-avoidance model variables on disability in patients with specific or nonspecific chronic low back pain. Spine (Phila Pa 1976).

[58] Rahman A, Reed E, Underwood M, Shipley ME, Omar RZ (2008). Factors affecting self-efficacy and pain intensity in patients with chronic musculoskeletal pain seen in a specialist rheumatology pain clinic. Rheumatology.

[59] Wettstein Markus, Eich Wolfgang, Bieber Christiane, Tesarz Jonas (2018). Pain Intensity, Disability, and Quality of Life in Patients with Chronic Low Back Pain: Does Age Matter?. Pain Medicine.

[60] Shiri R, Karppinen J, Leino-Arjas P, Solovieva S, Viikari-Juntura E (2010). The association between smoking and low back pain: a meta-analysis. Am J Med.

